# Paroxysmal epigastric pain 30 years after abdominal trauma surgery—a case report

**DOI:** 10.3389/fsurg.2023.1182171

**Published:** 2023-05-30

**Authors:** Yisheng Zhang, Xin Lu, Yiyao Xu

**Affiliations:** Department of Liver Surgury, Peking Union Medical College Hospital, Beijing, China

**Keywords:** trauma surgery, epigastric pain, reconstruction, duodenal dilatation, choledocholithiasis

## Abstract

A 58-year-old male was admitted to the liver surgery ward of Peking Union Medical College Hospital as the result of recurrent cholangitis in the past six months. Preoperative abdominal CT and gastrointestinal radiography showed duodenal dilatation and reconstruction of gastrointestinal tract, which might be related to the laparotomy and hemostasis performed due to traffic accident 30 years ago. The operative method of that surgery might be the reason for the patient's choledocholithiasis and duodenal dilatation.

## Presentation of case

A 58-year-old man was transferred to Peking Union Medical College Hospital for management of newly diagnosed cholangitis.

Six months before admission, the patient began to have episodes of epigastric pain after meal, without nausea, vomiting or diarrhea, the pain could be relieved after rest. Three months previously, he was hospitalized in the local emergency department because of the aggravation of postprandial epigastric pain and fever. The highest body temperature could reach 38.7°C. Choledocholithiasis was diagnosed by abdominal CT in local hospital. He was treated with fluid replacement and antibiotic therapy for a week until symptoms relief. One week before admission, severe epigastric pain with fever and chills occurred, and the highest body temperature could reach 39.0°C. The patient had anorexia and fatigue, but did not lose weight through the course of the disease. Treated with antipyretic and antibiotic medication, he was transferred to Peking Union Medical College Hospital for further treatment.

The patient underwent exploratory laparotomy for hemostasis 30 years ago because of a traffic accident. Abdominal incisional hernia was developed after laparotomy and detailed medical records of that operation were not available. He did not have hypertension, coronary heart disease, viral hepatitis or exposure to tuberculosis (timeline of the patient medical records is demonstrated in the Table 1).

**Table 1:** A brief timeline of the patient's medical records from 30 years before this hospitalization to outpatient follow-up 3 months after this surgery.

Timeline	Events & manifestations	Interventions
30 years before admission	Intraperitoneal hemorrhage caused by traffic accident	Hemostasis by laparotomy.
6 months before admission	Paroxysmal postprandial epigastric pain	Symptoms alleviated voluntarily.
3 months before admission	Postprandial epigastric pain and fever	With fluid replacement and antibiotic therapy (diagnosed as choledocholithiasis), symptoms could be alleviated.
1 week before admission	Severe epigastric pain with fever, chills, anorexia and fatigue	With antipyretic and antibiotic medication, symptoms could not be relieved.
		Transferred to Peking Union Medical College Hospital
1 month post operation	Mild abdominal discomfort and postprandial abdominal distension	Patient was instructed to eat semi-liquid foods, have more meals a day but less food at each, and use gastrointestinal motility drugs.
3 months post operation	Substantially back to the normal diet without obvious discomfort symptoms	Regularly follow up at the outpatient clinic in the future.

On physical examination, the temperature was 36.0°C, the heart rate 73 beats per minute, the respiratory rate 17 breaths per minute, the blood pressure 109/67mmHg and the oxygen saturation 99 percent while the patient breathing ambient air. No significant yellowing was found in the skin or mucous membrane of the patient. His abdomen was flat and longitudinal old surgical scar and drainage tube scar could be seen. The abdominal wall herniation could be found on the left side of the incisional scar and the hernia sac was flexible and painless. Superficial lymph nodes of the patient could not be touched. The upper abdominal tenderness and rebound pain were positive, with negative Murphy's sign and negative shifting dullness. Liver and spleen could not be reached under the rib. The remainder of the examination was normal.

On the day 1 of admission, laboratory test indicated sodium 132 mmol/L, potassium 4.8 mmol/L, chloride 94 mmol/L, calcium 2.48 mmol/L, carbon dioxide 23.7 mmol/L, urea 3.39 mmol/L, creatinine 65 µmoI/L, albumin 43 g/L, direct bilirubin 6.6 mmol/L, total bilirubin 9.8 mmol/L, serum alanine aminotransferase 102 U/L, hemoglobin 129 g/L, white cells 9,410 per mm^3^, platelets 558,000 per mm^3^, alpha fetoprotein 4.9 ng/ml, carcinoembryonic antigen 3.8 ng/ml, carbohydrate antigen 19-9 426.6 U/ml.

## Preoperative examination

**Figure 1:**
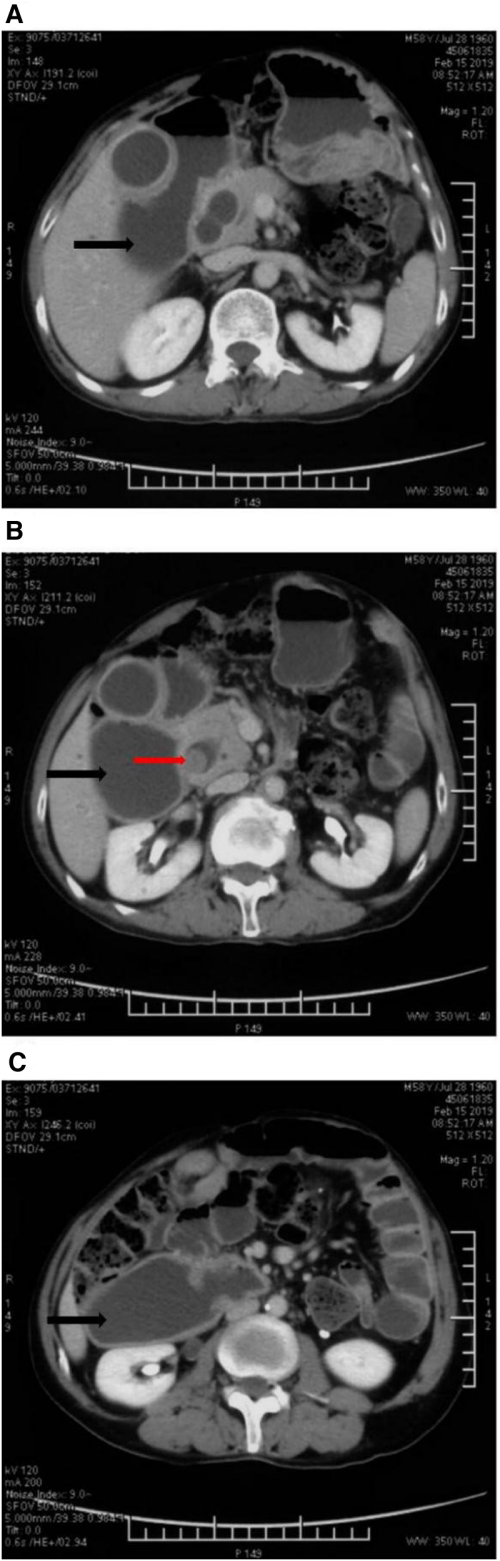
Abdominal enhanced CT images: the dilated duodenum was indicated by black arrows in (**A**), (**B**) and (**C**). The stone in the dilated common bile duct was marked with a red arrow in (**B**).

The patient was admitted to the liver surgery ward of Peking Union Medical College Hospital. He received abdominal CT scan again to confirm the diagnosis and assess the currant state of the illness. The examination results demonstrated multiple hepatic hemangioma, choledocholithiasis, exterior and interior cholangiectasis of liver, abdominal hernia on the left side and unexpectedly, significantly dilated duodenal cavity (Figure 1).

Duodenal dilatation is not a common manifestation of choledocholithiasis (1). In general, duodenum dilatation can be caused by physiologic and pathologic changes such as congenital megaduodenum, torsion of duodenum or small intestine, mechanical obstruction caused by benign and malignant tumor in abdominal cavity, intestinal stenosis due to anabrosis cicatrices, and there are even rare causes such as parasitic infections and superior mesenteric artery syndrome (Wilkie's syndrome) (2–5).

The patient's symptoms of paroxysmal abdominal pain appeared half a year ago and worsened recently. Carbohydrate antigen 19-9 increased to 426.6 U/ml but abdominal CT did not show any space-occupying lesions except choledocholithiasis. The rise in carbohydrate antigen 19-9 might be explained by inflammation within the bile duct but the etiology of duodenal dilatation in this case remained unclear (6).

**Figure 2:**
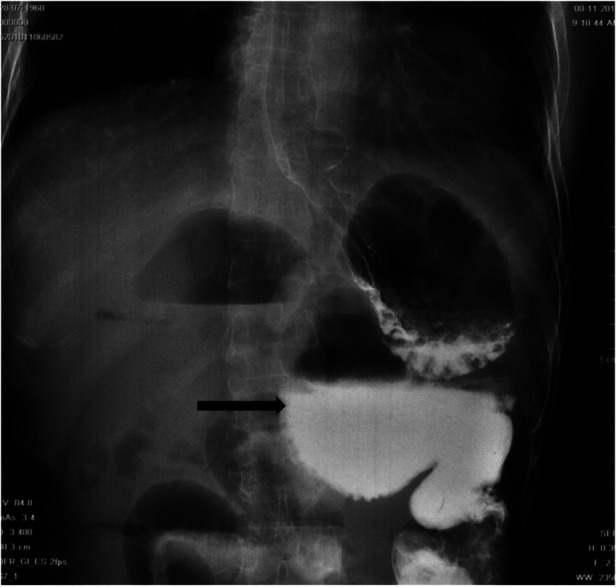
X-ray gastrointestinal radiography image: Ten minutes after taking barium, a large amount of contrast medium remained in the dilated duodenal cavity. The blind end of the duodenum filled with contrast medium was indicated by the black arrow.

Therefore, in order to further clarify the cause of the disease, the patient underwent gastrointestinal radiography with barium. Observed by x-ray, no obvious abnormality in esophagus was seen. However, once barium entered the gastric, it flowed along two directions. In one direction, the filling of contrast medium was not obstructed but in the other one, barium accumulated in the intestine and demonstrated the enteric cavity dilatation (Figure 2). This dilated lumen was consistent with the CT findings of duodenal dilatation and the gastrointestinal diversion might caused by the abdominal trauma surgery 30 years ago. As the medical records of that surgery were not available, the intraoperative condition and the specific procedures could not be known. It could be inferred from the results of CT and gastrointestinal radiography that a portion of the duodenum was closed to form a blind end and the distal intestine was utilized for gastrointestinal anastomosis.

To alleviate symptoms, the patient had strong desire to receive surgical treatment upon admission. Considering that the symptoms of cholangitis were still repeated and progressively aggravated after conservative treatment, this case was in accordance with rationale and indications for common bile duct exploration. As the patient had a history of abdominal trauma surgery, intra-abdominal adhesion might be unaffordable for laparoscopic surgery. This operation was tentatively for laparotomy exploration, cholecystectomy and common bile duct exploration.

## Surgical record

**Figure 3:**
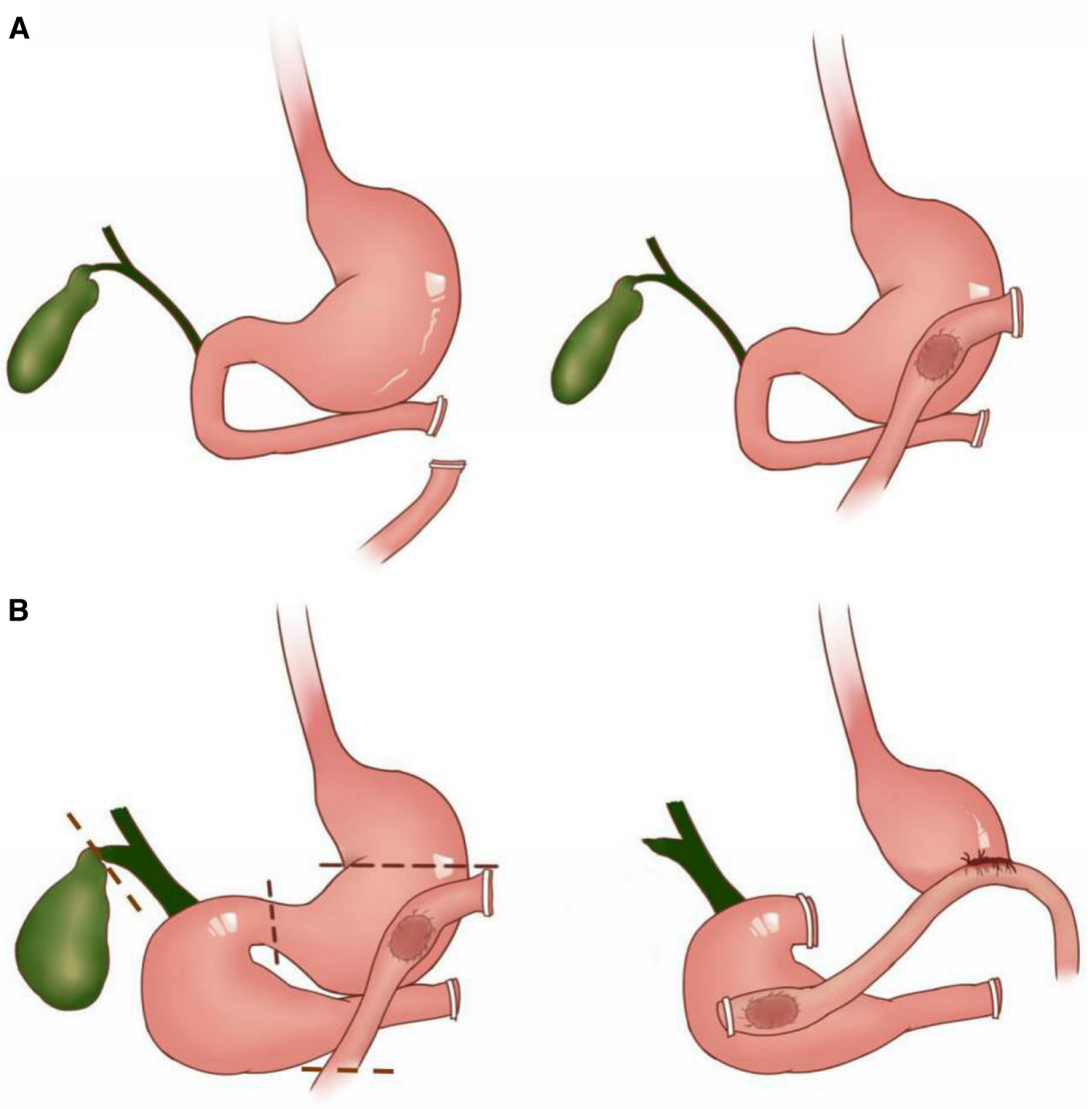
Schematic diagram of two abdominal operations for the patient: (**A**) demonstrated the presumed operation method of the abdominal trauma surgery 30 years ago. The distal end of the duodenum was cut off and closed. The lateral jejunum and the antrum were anastomosed sideways. (**B**) Showed the surgical strategy of the operation for removal of common bile duct stones and relief of duodenal dilatation. The duodenal cavity, gallbladder and extrahepatic bile duct were significantly dilated compared with the normal state. The dotted lines represented the sites where the incision and closure operations were performed during the surgery. Jejunum was anastomosed with the lateral wall of duodenum and the posterior wall of stomach at 2 cm and 20 cm away from the jejunal incision respectively.

The patient was placed in supine position. Under general anesthesia, laparotomy exploration began and the original median abdominal incision was used to incisive the abdomen. The defect of rectus abdominis was found in the left abdominal wall. There was extensive adhesion of small intestine, mesentery and omentum in abdominal cavity. Tight adhesion between lesser curvature of stomach and left liver was observed. The gallbladder was significantly enlarged and its size was about 15 cm  ×  8 cm. The duodenum was markedly dilated and the intestinal wall was thickened and edematous. The end to side anastomoses between the upper jejunum and the greater curvature of the stomach could be seen after the chief surgeon dissociated the intra-abdominal adhesion. Treiz ligament and upper jejunum were not found during exploration. Combined with the history and preoperative examination, it was speculated that the distal duodenum was closed by the previous exploratory laparotomy as the result of traumatic rupture of duodenum, and the upper jejunum was anastomosed to the gastric antrum side to side (Figure 3A). Therefore, in addition to extract stones in common bile duct, this operation also planned to relieve the problem of duodenal stasis and reflux.

After the chief surgeon dissociated the hepatoduodenal ligament, the enlarged common bile duct, which was about 2 cm in diameter and thickened in the wall was revealed. Cholecystectomy by conventional method and exploration of ductus choledochus by choledochoscope were performed. Stone with a diameter of 1.5 cm could be seen in the lower segment of the common bile duct. After the stone was completely removed with a stone basket, the common bile duct and the left and right hepatic ducts were re-explored with choledochoscope, and no stone was found.

The jejunum was cut 15 cm away from the original gastrointestinal anastomosis, and the right gastric and gastroepiploic arteries and veins were cut off near the gastric wall. The hepatogastric ligament and the remnant omentum were cut off, and the stomach was free to duodenal bulb. The duodenum was cut off 1 cm away from the distal pylorus, and the gastric body was cut off at 2 cm near the original gastrointestinal anastomosis. After finishing these procedures, the distal stomach, the original gastrointestinal anastomosis and the initial segment of jejunum were completely removed for pathological examination. The jejunum was lifted 20 cm away from the incision and the side wall was anastomosed with the posterior wall of the stomach. Jejunum-duodenum side to side anastomosis was performed at the horizontal part of the dilated duodenum and 2 cm away from jejunal incision (Figure 3B). On scrupulously inspection, the anastomotic stomas were unobstructed without tension, and there was no active bleeding in abdominal cavity.

## Follow-up information

Postoperative intravenous rehydration, nutritional support, acid suppression and anti-infection therapy were utilized, and the patient recuperated stably and had no surgical/nursing complication. On the day10 after the surgery, the patient was discharged smoothly after pulling out the abdominal drainage tubes and removing the wound sutures. The follow-up information of this patient at the outpatient clinic at 1 month and 3 months post-operation were recorded as follows.

1 month after surgery: the patient felt that the pain in the epigastrium had almost alleviated, and only slight discomfort was felt in the surgical area. Meanwhile, the patient complained of having mild postprandial abdominal distension. So gastrointestinal motility drugs were suggested to use for 2 weeks and the diet of semi-liquid foods with multiple-meal-with-small-amount-for-each was recommended. Laboratory blood test demonstrated: sodium 145 mmol/L, potassium 3.9 mmol/L, chloride 108 mmol/L, calcium 2.13 mmol/L, carbon dioxide 23.0 mmol/L, urea 6.71 mmol/L, creatinine 78 µmoI/L, albumin 33 g/L, direct bilirubin 3.7 mmol/L, total bilirubin 11.4 mmol/L, serum alanine aminotransferase 6 U/L, hemoglobin 99 g/L, white cells 8,820 per mm^3^, platelets 189,000 per mm^3^, alpha fetoprotein 4.4 ng/ml, carcinoembryonic antigen 5.2 ng/ml, carbohydrate antigen 19-9 21.3 U/ml. The postoperative pathology of the patient showed chronic cholecystitis and chronic inflammation of small intestinal mucosa, without any malignant manifestations.

3 months after surgery: the patient stated that currently there was almost no abdominal discomfort symptom and postprandial bloating or diarrhea was not observed when consuming a normal diet. The regions of surgery were visualized clearly in abdominal CT. The patient was very satisfied with the operation effects and postoperative recovery. As he is not a patient with malignant tumor, a favorable prognosis is predicted, and it is recommended to follow up regularly at the outpatient clinic every year.

## Management discussion

Gastrointestinal anastomosis by abdominal trauma surgery 30 years ago resulted in duodenal fluid, bile and pancreatic juice flowing back into the stomach through the pylorus. It could be the reason for the dilation of duodenum, common bile duct and gallbladder. The formation of choledocholithiasis may also be related to cholestasis caused by that operation. In some degree, the reconstruction strategy of the gastrointestinal tract in that operation performed 30 years ago, have some similarities with the Billroth II reconstruction. They both divide the digestive tract downstream of the stomach into an afferent loop and an efferent loop. In this case, the dilatated biliary tract and duodenum can be treated as the the afferent loop, and the alimentary canal sutured on gastric antrum can be treated as the efferent loop. The common manifestation of the afferent loop outlet obstruction after Billroth II reconstruction is acute or chronic pancreatitis (7). The choledocholithiasis, dilatation of duodenum and biliary tract in this case, are rare manifestation of high gastrointestinal obstruction (8).

In emergency cases, in order to quickly perform hemostasis and deal with intestinal rupture, it is acceptable to perform an unconventional operation like that, especially considering the limitation of medical conditions and the influence of abdominal bleeding. But there are its limitations: (1) the activity of digestive enzymes may be affected by the change of pH value as duodenal fluid, bile and pancreatic juice backflow to the stomach. (2) The high pressure of duodenal blind end may lead to intestinal fistula after operation. (3) The accumulation of digestive juice can cause the expansion of duodenum, common bile duct and gallbladder. (4) Cholestasis may cause the formation of gallstones and bile duct stones. In summary, continuous follow-up observation is needed for patients who have undergone unconventional surgical treatment, and the necessity for further treatment needs to be evaluated based on the clinical manifestations.

## Data Availability

The original contributions presented in the study are included in the article, further inquiries can be directed to the corresponding author.
